# Supplementary material for: Can forensic radiological skeletal age estimation be performed by examining ischiopubic-ilioischial-iliopubic synchondrosis in computed tomography images?
Source: PLoS One. 2022 Apr 28;17(4):e0266682. doi: 10.1371/journal.pone.0266682 (PMC9049324; doi:10.1371/journal.pone.0266682)
Supplement: Attachment — Submitted filename: responses to criticism 26102021.docx [file pone.0266682.s002.docx]

Reviewer 1:

- In the abstract, why the 'Instrument and methods' was used? Is there any instrument?

- The "instrument" phrase has been removed and corrected as "Methods."

- Please give some more pictures as examples of the CT images showing various stages of fusion of the synchondrosis.

- New images are added.

- Please explain more methods in the abstract.

- Added more descriptive information to the Methods section.

- "Two hundred sixty-three children (118 girls and 145 boys) between the ages of 8 and 16 without any health problems participated in the study"- this is a part of methodology and not the results.

- This part is transferred to the methodology section.

- Why are you mentioning "Discussion and results', why not 'Results and Discussion'. Please use standard format given by the journal.

- The "Result" statement was removed from the abstract section and the "Conclusion" statement was added instead.

- Please change '8 and 16' to '8 and 16 years'

- ok

- "Age is one of the physical features, along with gender,.... Age is not a feature?

- This part is removed.

- Please replace gender with 'sex'. Please use appropriate words throughout as the inappropriate words may change the meaning of the sentences.

- The word "gender" has been replaced with the word "sex."

- "According to the defined UNICEF age limits,"

- This part is removed.

- Please mention dates/years of the examination of the patients.

- ok

- Please discuss the sex differences as obtained in the study in light with other studies.

- New additions were made to the discussion.

- Why are you mentioning 'results' in stead of 'conclusion'?

- The "Result" statement was removed from the abstract section and the "Conclusion" statement was added instead.

- In the reference section, The format of the references is not uniform, please use journal's guidelines for the same. Please do not use papers from predatory journals such as reference no. 6, 13 etc.

- These references were removed and new ones were added in their place.

- The introduction is very superficial, please write detailed introduction.

- Additions were made by detailing the introduction section.

- The conclusion should contain concrete findings of the study according to the title as well the objective of the study. Also mention, What is the percentage of accuracy of the determination of age from these dimensions for the mentioned ages?

- The conclusion section was arranged to be more consistent with the findings.

- In the reference section, The format of the references is not uniform, please use journal's guidelines for the same.

- References were reviewed according to journal guidelines.

Reviewer 2

Reviewer #2: Thank you for presenting this significant study for forensic age estimation.

Please find below some suggestions to help improve the article.

- The language needs to be evaluated in further detail. Please use consistent tense forms.

- The results needs to be further elaborated. What were the significance values for the Chi-square tests. The p-values should be correlated along with the significance values.

OK

- Discussion should focus on comparison with other studies and their findings and discussing the relevance of similarities and differences in these studies.

With new references, the discussion has been expanded upon your suggestion.

- Most of the presently submitted discussion section, including validating Age estimation as well as CT studies, legal provisions, ability of pelvis to withstand trauma, etc should all be detailed in the Introduction and referenced in brief in discussion, if required.

With the new resources, the introduction part has been expanded according to your suggestions.

Reviewer 3

reviewer #3: The manuscript is technically sound with appropriate statistical analysis. Manuscript is in good standard English. Data underlying the findings in the manuscript is fully available. Please do strict in research ethics.

Ethics committee approval document is attached.
